# A rare case of adult ovarian hernia in MRKH syndrome

**DOI:** 10.1259/bjrcr.20160080

**Published:** 2017-05-06

**Authors:** Himansu Shekhar Mohanty, Kapil Shirodkar, Aruna R Patil, Navin Rojed, Govindrajan Mallarajapatna, Shrivalli Nandikoor

**Affiliations:** Department of Radiology, Apollo Hospitals, Bangalore, India

## Abstract

Inguinal hernias containing ovary have a documented incidence of 3%. Most of the cases are associated with congenital anomalies of genital tract such as Mayer-Rokitansky-Küster-Hauser (MRKH) syndrome. A 20-year-old female presented with primary amenorrhoea, normal secondary sexual characteristics and genetic karyotyping showing 46XX chromosome. On USG abdomen and pelvis examination complete absence of uterus, cervix and vagina was found. Both the ovaries were seen away from normal anatomical location in bilateral inguinal canal without significant complication. MRI study confirmed agenesis of uterus, cervix and vagina; bilateral inguinal hernia with ovaries as contents. Type 1 MRKH syndrome with bilateral ovarian hernias was diagnosed.

## Background

Inguinal hernia is the most common hernia occurring in adult population. It is relatively uncommon in female and occurs in less than 5% of females.^1^ In adult females, indirect hernias occur more frequently than direct hernias and are typically seen during the 4th–6th decade.^1^ Mostly inguinal hernial sac contains omentum or small bowel but caecum, appendix and sigmoid colon are seen at times and urinary bladder may also protrude as content.^2^ Inguinal hernias containing ovaries (ovarian hernias) occur very rarely in adult females (approximately <3%).^3^ Ovarian hernia is not uncommon in female infants and paediatric patients and often associated with congenital genitourinary tract anomalies such as Mayer-Rokitansky-Küster-Hauser (MRKH) syndrome.^4–6^ Complications of ovarian hernia includes ovarian torsion, incarceration or salpingitis.

The prevalence of MRKH syndrome is 1 in 4,000 to 1 in 5,000 female patients and its association with inguinal hernia containing ovarian tissue is a rare condition with very few cases reported in literature.^7^ We report a case of MRKH syndrome associated with ovarian inguinal hernia with review of relevant medical literature.

## Case report

**Figure 1.**
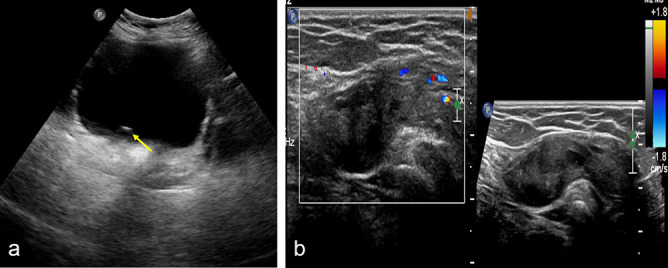
(a) USG pelvis showing absence of uterus, cervix and vagina with distended urinary bladder and right ureteroceles (yellow arrow). (b) USG grey scale and Doppler study images showing the right ovary in inguinal canal with patent vascularity and normal ovarian follicles inside.

A 20-year-old female with history of primary amenorrhoea referred to our radiology department for further evaluation. On physical examination, all the secondary sexual features were well made out. In view of primary amenorrhoea, an abdominal sonography was performed which showed upper abdominal organs in normal anatomical location. On pelvic sonography, there was complete non- visualization of uterus, cervix and vagina (Figure 1a). Both the ovaries were not seen in their normal anatomical location and on extensive search, they were localized in bilateral inguinal canals (Figure 1b). On Doppler study there was no significant vascular compromise in both the ovaries. In addition to above findings bilateral ureteroceles were also noticed (Figure 1a).

**Figure 2.**
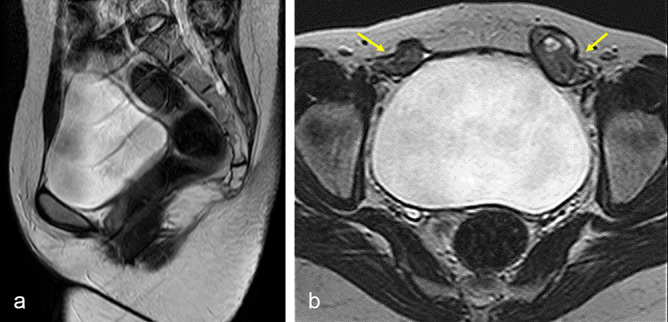
(a) *T*_2_ weighted sagittal section of pelvis showing complete absence of uterus, cervix and vagina with normal appearing urinary bladder. (b) *T*_2_ weighted axial image of pelvis showing both the ovaries in bilateral inguinal canal (yellow arrows).

**Figure 3.**
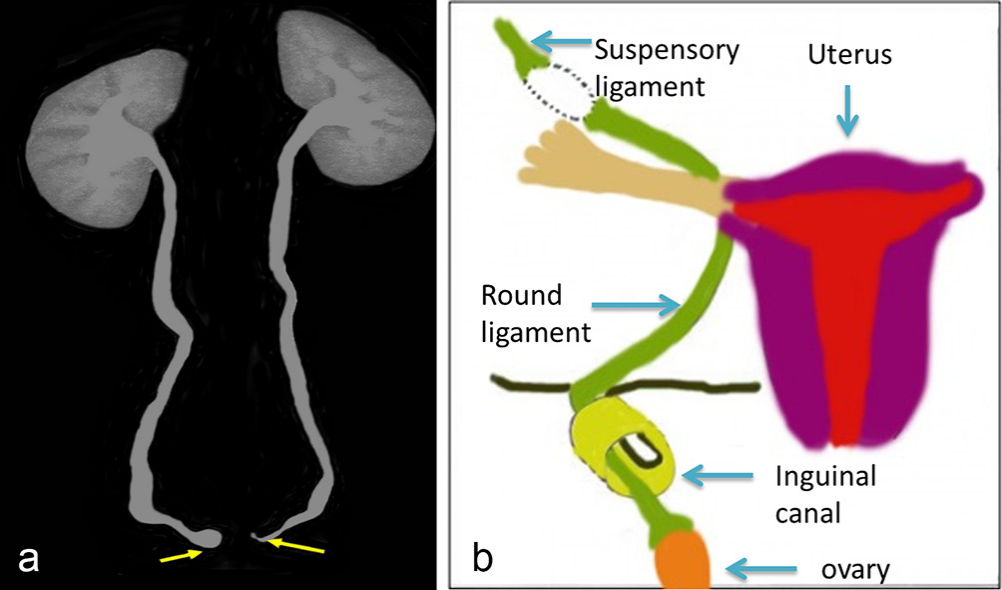
(a) Delayed contrast-enhanced CT abdomen and pelvis in coronal section showing normal excretion of contrast to urinary bladder with bilateral ureteroceles (yellow arrows). Both the kidneys are located in normal anatomical location. (b) The diagram showing the inguinal canal, hernia sac with ovary inside the sac.

CT urogaphy and screening MRI pelvis were advised for detailed evaluation of urogenital anomalies which confirmed complete agenesis of the uterus, cervix and vagina (Figure 2a). Both the ovaries were visualized in bilateral inguinal canals (Figure 2b). In abdomen the kidneys were normal in size, morphology and located in normal anatomical location without any developmental anomalies (Figure 3a). Delayed CT KUB scan showed bilateral ballooning of vesicoureteric junction confirming the bilateral ureteroceles (Figure 3a). Genetic karyotyping showed 46XX chromosome pattern.

The diagnosis of Type I MRKH syndrome with bilateral ovarian hernias was established.

## Discussion

### Anatomy of the inguinal canal

Inguinal canal is an oblique passage in the lower part of abdominal wall measuring approximately 4 cm in length that is lined by the aponeuroses of three muscles: the external oblique, internal oblique and transversus abdominis muscles.

In females, the inguinal canal transmits the round ligament of the uterus and the ilioinguinal nerve to the labia majora, a vein and an artery from the uterus that forms a cruciate anastomosis with the labial arteries. In both genders, the canal also contains lymphatic vessels and sympathetic nerve fibres, along with fat and connective tissue

Entrapment of the female adnexa may occur in inguinal canal. It is found more frequently in infants owing to anatomical causes including (a) a relatively short inguinal canal, (b) a canal that has an oblique direction through the abdominal wall and (c) a diverticulum of Nuck, a peritoneal pocket associated with the round ligament that corresponds to the vaginal processus in the male infant.^8,9^

### Mechanism of ovarian hernia

An anatomical abnormality with primary weakness of the uterine and ovarian suspensory ligaments is suspected. Thomson offered the hypothesis that if there is failure of fusion of the Mullerian ducts leading to excessive mobility of the ovaries plus non-fusion of the uterine cornua, the chance of herniation of the entire uterus, ovary and fallopian tube into the inguinal canal is increased.^10^ On the other hand, Fowler theorized that elongated ovarian suspensory ligaments were the primary cause or the secondary effect of a hernia.^11^

T. Okada et al.^9^ suggested that weakness of the broad ligaments or ovarian suspensory ligaments can contribute to ovarian herniation into the inguinal ring, which exaggerates on increased intra-abdominal pressure.

Further risk factors may include lengthening of the broad ligament, uterine or ovarian suspensory ligaments in high parity patients resulting in displacement of adnexal structures and high intra-abdominal pressures from frequent valsalva maneuvers in patients with chronic cough or frequent heavy lifting.^9^

### Mayer-Rokitansky-Küster-Hauser syndrome

Mayer–Rokitansky–Küster–Hauser (MRKH) syndrome refers to congenital aplasia of the uterus and the upper two-thirds of the vagina in females with normal ovaries and fallopian tubes, secondary sexual characteristics and 46XX karyotype. MRKH syndrome is classified into two types based on associated anatomical features. It was earlier considered as sporadic but now the theory of being autosomal dominant has been incorporated. The incidence is 1 out of 4500 females.^12^

*Type I MRKH syndrome* or Rokitansky sequence is usually isolated type while *Type II MRKH syndrome (*MURCS association or genital renal ear syndrome) is associated with renal, vertebral, and to a lesser extent, auditory and cardiac defects. The young females present with normal female type body with the normal functioning ovaries. The external genitalia do not show any abnormality as per the anatomical lay out. The patients usually have normal thelarche and adrenarche. Ovarian torsion and infarction are known complications in 2–33% of the patients presenting as non-reducible groin swellings.^13^

## Imaging features

The normal ovary in the hernia sac (Figure 3b) appears hypoechoic with multiple variable-sized sonolucent cysts (Figure 1b). In case of torsion, the ovary appears bulky with multiple peripherally arranged follicle and areas of haemorrhage within. On colour Doppler, in early stages there will be preserved peripheral vascularity which in the later stages disappears with total absence of colour flow. Furthermore, transabdominal sonographic scans of the pelvis may reveal the absence of normal ovary in the lower pelvis.

*T*_2_ weighted sequences are useful sequences in female pelvic imaging owing to the ability to demonstrate the zonal anatomy of the uterus. Sagittal *T*_2_ weighted spin echo and oblique long axis *T*_2_ weighted fast-spin echo images obtained parallel to the long axis of the uterus can be used for diagnosis of uterovaginal anomalies. Uterine hypoplasia or agenesis is best diagnosed on *T*_2_ weighted sagittal images (Figure 2a). Normal vagina is seen as a tube of intermediate signal intensity between the base of bladder and urethra anteriorly and the anal canal posteriorly. Vaginal agenesis is best demonstrated on axial images of MRI.^14^

Similar case report was described by Omari et al in a 31-year-old female with MRKH syndrome with a solitary right pelvic kidney and right utero-ovarian inguinal hernia.^15^

## Differential diagnosis

The differentials to be considered include^16^

- Imperforate hymen (absent fringe of hymenal tissue)
- Transverse vaginal septum (normal hymen with proximally obstructed vaginal canal)
- Androgen Insensitivity syndrome (presence of testes with androgenic hormones with normal breast development and absent secondary sexual characteristics)
- 17α-hydroxylase deficiency (phenotypically normal female external genitalia, a blind short vaginal pouch, no uterus or fallopian tubes, and dysgenetic intra- abdominal testes)
- congenital absence of the vagina (with or without uterine structures)

### Management

The management of MRKH is multipronged with treatment aimed at psychological, medical and surgical management. Psychological counselling is provided to alleviate the emotional effects. Healthy sexual relationships and genetic offspring through *invitro* fertilization and surrogacy are possible. Non-surgical creation of the vagina is the most common approach with manual daily self-dilation of the vaginal dimple. Anatomic and functional success by vaginal dilation was reported in upto 90–95% patients in studies done by Jones et al and Roberts et al.^17,18^

Surgery is considered in patients with failure of manual self-dilatation or in patients who prefer surgical creation of a vaginal canal to allow sexual intercourse. Surgical intervention in a timely fashion may also be necessary in order to prevent and relieve torsion and to return normal perfusion to the adnexa and prevent subsequent infertility. A number of surgical techniques can be used to create a neovagina such as McIndoe technique, William vaginoplasty, Rotational flap procedure, Intestinal neovagina and Vacchietti technique.^19^ Other techniques, such as bowel graft neovagina, use of buccal mucosa, amnion and various other allografts, are less commonly applied to females with müllerian agenesis.^16^ Recently a new procedure was described by Benedetti Panici et al in which vaginoplasty was done with modified Abbè-McIndoe technique with autologous *in vitro* cultured vaginal tissue.^20^

Regular pelvic examinations to rule out vaginal stricture or stenosis as well as the use of condoms to prevent sexually transmitted diseases is recommended. Routine vaginal cytologic testing and human papillomavirus vaccination is not recommended.^16^

## Conclusions

In summary, we hereby present a rare case of Type I MRKH syndrome with bilateral uncomplicated indirect inguinal ovarian hernia in a pre-menopausal adult female. Multiple imaging studies may be necessary to assist in diagnosis, including ultrasound and/or cross-sectional imaging by MRI. Torsion and subsequent infarction may result in an unsalvageable ovaries leading to subsequent infertility. Proper surgical and medical management is required to ensure preservation of fertility especially in young premenopausal females.

## Learning points

1. Bilateral ovarian inguinal hernias are rare occurrences in female.
2. Association of MRKH syndrome with ovarian hernias are not so uncommon.
3. Early diagnosis of ovarian hernias should be made to preserve the fertility.
4. Complications of ovarian hernia include torsion and infarction which should be suspected in appropriate clinical scenario.

## Consent

Informed consent to publish this case (including images) was obtained and is held on record.
